# Psychiatric outcomes and overall functioning in healthcare students during the first wave of the COVID-19 pandemic: a cross-sectional study

**DOI:** 10.47626/2237-6089-2021-0416

**Published:** 2023-06-27

**Authors:** Flávia de Moraes, Angelica de Baumont, Carolina Blaya Dreher, Gustavo Gauer, Gisele Gus Manfro

**Affiliations:** 1 Programa de Transtornos de Ansiedade Hospital de Clínicas de Porto Alegre Universidade Federal do Rio Grande do Sul Porto Alegre RS Brazil Programa de Transtornos de Ansiedade, Hospital de Clínicas de Porto Alegre, Universidade Federal do Rio Grande do Sul (UFRGS), Porto Alegre, RS, Brazil.; 2 Faculdade de Psicologia UFRGS Porto Alegre RS Brazil Faculdade de Psicologia, UFRGS, Porto Alegre, RS, Brazil.; 3 Programa de Pós-Graduação em Psiquiatria e Ciências do Comportamento UFRGS Porto Alegre RS Brazil Programa de Pós-Graduação em Psiquiatria e Ciências do Comportamento, UFRGS, Porto Alegre, RS, Brazil.; 4 Programa de Pós-Graduação em Psicologia UFRGS Porto Alegre RS Brazil Programa de Pós-Graduação em Psicologia, UFRGS, Porto Alegre, RS, Brazil.

**Keywords:** Anxiety, depression, quality of life, COVID-19 pandemic, students on health care courses

## Abstract

**Introduction:**

There is evidence that the coronavirus disease 2019 (COVID-19) pandemic impacted students on health care courses, including evidence of associations between anxiety or depression and inadequate coping mechanisms or unhealthy habits. However, little is known about possible predictors of mental health or psychiatric symptoms in Brazilian health care students during this period.

**Objective:**

To evaluate possible factors associated with anxiety and depressive symptoms, used to measure psychiatric outcomes, and quality of life, used as a parameter of overall functionality, in Brazilian students on health care courses during the COVID-19 pandemic.

**Methods:**

A cross-sectional study was conducted with Brazilian students on health care courses from May to December 2020. Participants were recruited through social media and answered a 71-item open online questionnaire exploring demographic characteristics and personal behavior during the pandemic, anxiety, depression, and quality of life. We searched for variables potentially associated with psychiatric symptoms and mental health in these individuals using Poisson regression models.

**Results:**

Multivariate models showed depression and anxiety were associated with poor quality of life and medication abuse was associated with greater anxiety and poor quality of life. Psychotherapy was an effective coping strategy for anxiety and meditation or mindfulness practice and physical activity improved the students’ quality of life.

**Conclusions:**

Our study presents important information about the factors associated with psychological impacts of the COVID-19 pandemic and strategies for coping with them that should be helpful to reflect on and for designing appropriate interventions.

## Introduction

In March 2020, the World Health Organization (WHO) declared the novel coronavirus disease 2019 (COVID-19) to be a pandemic,^1^ leading to anxiety, mental health distress, and physical health problems.^2 - 4^ At that time, there were no vaccines or medications with known efficacy to treat the condition, so mitigation actions such as social distancing were used across different countries, causing a significant and negative impact on the mental health of the general population, including the Brazilian population.^5 - 8^

Students on health care courses had a higher risk of exposure to coronavirus infection compared with the general population when attending their regular curricular internships in hospitals and at healthcare units.^9^ After completing 9 years of schooling (basic education, high school, and college), students that choose health care courses attend university for another 5 years within their areas of expertise (nursing, pharmacy, physiotherapy, and others), or for 6 years for medicine, after passing a national exam. During graduation, students are taught content that is defined by the curricular guidelines for their undergraduate courses, related to the individual health-disease process, public health, and community health, integrated with epidemiology and professional practice. Courses must cover theoretical and clinical practice dimensions, considering mainly inclusion of internships at hospitals, outpatient clinics, and in the primary care network. In the last 2 years of undergraduate study, activities basically involve direct practical activities and patient care.

As a measure to protect academics and reduce the risk of contagion among the population, face-to-face classes and internships were canceled at universities. Even so, many students on health care courses chose to continue their curricular internships, where possible, motivated by altruistic feelings or seeking recognition or increased knowledge.^10^ On the other hand, some students might feel afraid about contracting and spreading the virus when working on the front line against COVID-19.^11^

In this unprecedented situation, some evidence has emerged about the long-lasting impacts on the physical and mental health of students in general and students on health care programs, in particular nursing students.^12^ Some of these recent studies have shown associations between anxiety or depression and inadequate coping mechanisms or unhealthy habits among health care students during the pandemic.^7 , 13 - 16^ Among Brazilian students, there was high prevalence of stress, anxiety, and depression in this period,^12 , 17 , 18^ however, no difference was shown compared to the period before the pandemic.^17^ Some of these students reported alcohol.^5 , 12 , 18^ and substance abuse.^7 , 12^ However, some students were able to adopt physical activity as a method to improve positive emotions and mental well-being, offering protection against depression and anxiety and thereby improving their quality of life.^18^

Despite these findings, little is known about mental health in Brazilian health care students during the pandemic, or about possible associations with psychiatric symptoms and overall mental functionality in this population. Thus, this study aimed to conduct a survey-based assessment of possible variables associated with anxiety and depressive symptoms, used as measures of psychiatric outcomes, and quality of life, used as a parameter of overall functionality, in Brazilian students on health care courses during the COVID-19 pandemic.

## Methods

### Study design

This study was a cross-sectional online survey conducted among Brazilian health care students from May 2020 to December 2020. The study was performed with questionnaires administered using Google Forms, a free tool for online surveys offered by Google. The survey followed the Checklist for Reporting Results of Internet E-Surveys (CHERRIES), a checklist of recommendations to help authors ensure complete descriptions of Web-based surveys.^19^

The survey was based on the literature and all the technical functionality of the electronic questionnaire was tested by the researchers before the link with the questionnaire was made available. The items were not randomized or alternated. No identification of multiple entries was used. Only completed questionnaires were analyzed.

The sample size was estimated by PEPI-for-Windows (WINPEPI) v. 11.65, expecting a minimal difference of one point in the Generalized Anxiety Disorder 7-item (GAD-7) between distinct health care undergraduate courses, with a 5% significance level and 80% power, resulting in a total of 432 students.

### Participants

Students on health care courses in Brazil were recruited through social networks (WhatsApp, Instagram, and Facebook) and by sending an invitation to participate in the survey to e-mail groups so that they could answer the open-source online questionnaire (Supplementary Material S1, available online-only). A convenience sample was selected, because the students who answered the online survey were not representative of all Brazilian states. Participants were able to review and change their answers using a “back” button.

Participation was anonymous and voluntary, with no monetary incentive, and an electronic informed consent form was made available to each subject who agreed to participate after being given a detailed and clear description of the main purposes of the study.

### Inclusion and exclusion criteria

We included students on health care courses aged 18 or over living in Brazil and excluded students from other academic areas.

### Instruments

We developed a 71-item self-administered questionnaire (seven - 38 items per page) with seven pages, divided into two parts: first, a custom-designed questionnaire based on the CoRonavIruS Health Impact (CRISIS) Survey^20^ that explored demographic characteristics and personal or close social network exposure to COVID-19 as well as personal protective behaviors adopted during the pandemic. We included 12 questions extracted from CRISIS, evaluating living, health habits, worries, eating behavior, coping, and use of tobacco or other drugs. The CRISIS Survey is available in many languages, including Portuguese.^20^

The second part investigated anxiety and depressive symptoms, used to measure psychiatric outcomes, and quality of life, used as a parameter of overall functioning, incorporating the following instruments:

- The Patient Health Questionnaire-9 (PHQ-9): is a self-administered 9-item scale with four-point response options^21 - 23^ that evaluates depressive symptoms. It has good internal consistency ( *α* =.87) and is validated in Portuguese for detecting depression.^23^
- GAD-7: this consists of seven items with a four-point response scale for assessing, diagnosing, and monitoring anxiety symptoms.^24 - 26^ It has a Cronbach coefficient of 0.916 and a reliability coefficient of 0.909 and the Brazilian Portuguese version of the GAD-7 is considered adequate for assessing symptoms of generalized anxiety disorder in Brazilian adults.^25^
- The Quality of Life and Satisfaction Questionnaire (QLESQ): is a 16-item scale containing eight subscales. It has a Portuguese version, which serves to assess levels of satisfaction and pleasure during the last week in eight functional domains.^27 , 28^ All Q-LES-Q subscales have a significant Cronbach’s alpha of 0.78 or greater.^27 , 28^

### Procedures

Participants answered the survey from May to December of 2020. A convenience sampling method was employed for data collection via social media with an online questionnaire including the instruments described above.

The survey link was posted on various university student platforms available on social media (e.g., Facebook). The study was entirely voluntary in nature and the participant could withdraw at any time without providing any justification. After respondents had read the informed consent form and agreed to it, the online questionnaire was released for completion.

A total of 462 respondents completed the survey and thus the final analysis could be carried out. No financial incentives were provided to participants and anonymity was maintained to ensure data confidentiality and reliability. This study was conducted online in full compliance with the provisions of the Declaration of Helsinki on research in human participants.

### Ethical considerations

This research was approved by the ethics committee at the Hospital de Clínicas de Porto Alegre (CAAE: 31873520.5.0000.5327) and conducted in accordance with the provisions of the Declaration of Helsinki. All consent forms, as well as the data collected, were treated as secret and confidential and were stored on a local electronic device, with all records in virtual or shared environments erased. Therefore, all documents are the sole responsibility of the main researcher.

### Data availability

Data supporting the findings of this study are available from the corresponding author upon reasonable request.

### Statistical analysis

All statistical analyses were performed using the Statistical Software for Social Sciences (SPSS) for Windows, version 21.0, and the level of significance was set at 0.05.

Sample characteristics were expressed as means (standard deviations [SD]), median (minimum and maximum), or percentages. We checked for normal distribution of quantitative data using the Kolmogorov-Smirnov test and by viewing histograms.

We tested associations with anxiety (GAD-7 ≥ 10) and depression (PHQ-9 ≥ 10) using chi-square tests for categorical variables and independent samples *t* tests or Mann-Whitney *U* sum tests for continuous variables. We also searched for variables associated with quality of life (QLESQ total scores) using *t* tests or Spearman’s correlations. Results of bivariate analyses were reported as means (SD) or percentages.

For multivariable analysis, we used Poisson regression with robust variance to model associations between anxiety (GAD-7 ≥ 10), depression (PHQ-9 ≥ 10), or quality of life (QLESQ total scores) and independent variables. We included independent variables in the model if p < 0.05 on bivariate analysis, or based on previous studies on mental outcomes during the pandemic.^4 , 10 , 11 , 29 - 33^

All independent variables in the multiple linear regression were tested for multicollinearity. Results are reported as prevalence ratios or beta and 95% confidence intervals (95%CI).

Additionally, attempting to understand whether participants from different undergraduate health care courses would differ in terms of mental health characteristics and disorders, as well as behaviors during the pandemic, participants were distributed into four groups according to their undergraduate course: nursing, pharmacy, medicine, and other health courses. We compared continuous variables between groups with one-way analysis of variance (ANOVA) or the Mann-Whitney *U* test and compared categorical variables with chi-square tests. Results were reported as means (SD) or percentages.

## Results

### Participants’ demographic and clinical characteristics

A total of 462 health care students were included, most were women (369 females; 79.9%) and ages ranged from 16 to 61 years. Most participants were from Rio Grande do Sul state (78.3%), a majority were residents of urban areas (66.9%), and only 16.2% reported living alone. The most prevalent academic category in our sample was medical students (42.6%) and 72.1% of all samples were interns at hospitals or at healthcare units.

The proportion of students with moderate to severe anxiety was 39.4% (GAD-7 ≥ 10) and 51.5% reported depressive symptoms (PHQ-9 score ≥ 10) ( Table 1 ). More detailed information on the demographic and COVID-19-related characteristics of the participants is summarized in Table 1 .

**Table 1:** Descriptive data on sample characteristics

Variable	
Female gender, n (%)	369 (79.9)
Age, median (range)	23 (16-61)
State, n (%)	
Rio Grande do Sul	362 (78.3)
São Paulo	40 (8.7)
Other states	60 (13.0)
Undergraduate course, n (%)	
Nursing	80 (17.3)
Pharmacy	84 (18.2)
Medicine	197 (42.6)
Other courses	101 (21.9)
Living alone, n (%)	75 (16.2)
Is interning, n (%)	333 (72.1)
Place of residence, n (%)	
Urban	309 (66.9)
Rural	153 (33.1)
Substance use, n (%)	
Alcohol	133 (28.8)
Medication	67 (14.5)
Other substances	29 (6.3)
Anxiety, n (%)	
GAD-7 < 10	280 (60.6)
GAD-7 ≥ 10	182 (39.4)
Depression, n (%)	
PHQ-9 < 10	224 (48.5)
PHQ-9 ≥ 10	238 (51.5)
Quality of Life (QLESQ) score, mean (SD)	43.92 (8.58)

GAD-7 = Generalized Anxiety Disorder 7-item; PHQ-9 = Patient Health Questionnaire-9; QLESQ = Quality of Life and Satisfaction Questionnaire; SD = standard deviation.

### Psychiatric outcomes

Presence of severe anxiety (GAD-7 ≥10) was associated with low quality of life, depressive symptoms, use of medication as a coping strategy (with a medical prescription), alcohol, medication abuse (unprescribed) as a coping strategy, and severe fear of the infection in bivariate analyses. Physical activity, living in urban areas, and no fear of contagion were associated with a lower frequency of anxiety (Table S1, available as online-only supplementary material). Being female, being older, having severe anxiety, living in rural areas, being on medication with a prescription or having psychotherapy, using alcohol as a coping strategy, and severe fear of COVID-19 were associated with depressive symptoms. Physical activity and higher quality of life were associated with a lower frequency of depression (Table S2).

Multivariate Poisson regression analysis using the presence of anxiety as the outcome showed that participants from undergraduate nursing courses had a higher prevalence (35.2%) of anxiety in comparison to medical students. Pharmacotherapy abuse was associated with a 31.6% higher prevalence of anxiety and depression was associated with a four times greater prevalence of anxiety. Psychotherapy and good quality of life were associated with a lower prevalence of anxiety symptoms ( Table 2 ).

**Table 2:** Multivariate Poisson regression model for anxiety in health care students (n = 462)

		95%CI		
			
Parameter	Exp (B)*	Lower	Upper	p
Intercept	0.344	0.144	0.823	0.016^†^
Undergraduate course				
Other courses	0.710	0.515	0.978	0.036^†^
Pharmacy	1.130	0.868	1.472	0.364
Nursing	1.352	1.072	1.705	0.011^†^
Medicine	1	-	-	-
Gender				
Female	1.067	0.827	1.378	0.618
Male	1	-	-	-
Is interning				
No	1.088	0.873	1.356	0.454
Yes	1	-	-	-
Living alone				
Yes	1.137	0.883	1.464	0.320
No	1	-	-	-
Fear of contagion				
Severe fear	0.624	0.364	1.069	0.086
Moderate fear	0.913	0.645	1.293	0.608
Little fear	0.988	0.797	1.224	0.910
No fear	1	-	-	-
Coping strategies				
Diaphragmatic breathing				
Yes	0.949	0.748	1.205	0.669
No	1	-	-	-
Physical activity				
Yes	1.021	0.794	1.314	0.871
No	1	-	-	-
Medication				
Yes	0.889	0.696	1.137	0.349
No	1	-	-	-
Psychotherapy				
Yes	0.757	0.594	0.966	0.025^†^
No	1	-	-	-
Substance use				
Alcohol				
Yes	1.169	0.959	1.424	0.122
No	1	-	-	-
Medication				
Yes	1.316	1.042	1.663	0.021^†^
No	1	-	-	-
Place of residence				
Rural	1.108	0.903	1.360	0.325
Urban	1	-	-	-
Depression				
PHQ-9 ≥ 10	4.270	2.843	6.412	< 0.001^‡^
PHQ-9 < 10	1	-	-	-
Quality of life				
QLESQ	0.976	0.960	0.993	0.005^§^

95%CI = 95% confidence interval; PHQ-9 = Patient Health Questionnaire-9; QLESQ = Quality of Life Enjoyment and Satisfaction Questionnaire.

Anxiety was defined as a Generalized Anxiety Disorder 7-item (GAD-7) score ≥ 10.

* Prevalence ratio.

^**†**^ p < 0.05; ^**‡**^ p < 0.001; ^**§**^ p < 0.01.

In a multivariate analysis using depressive symptoms as the outcome, medication use was associated with a 28.1% higher prevalence of depression, psychotherapy with a 23.8% higher prevalence, and presence of anxiety was associated with twice the prevalence of depression. Moderate fear in comparison to severe fear, older age, and higher quality of life were all associated with lower prevalence of depression ( Table 3 ).

**Table 3:** Multivariate Poisson regression model for depression in health care students (n = 462)

		95%CI		
			
Parameter	Exp (B)*	Lower	Upper	p
Intercept	4.44	1.842	10.701	0.001^†^
Undergraduate course				
Other courses	1.148	0.936	1.408	0.185
Pharmacy	0.95	0.761	1.185	0.647
Nursing	0.922	0.76	1.119	0.411
Medicine	1	-	-	-
Age	0.977	0.959	0.995	0.015^‡^
Gender				
Female	1.08	0.859	1.358	0.509
Male	1	-	-	-
Is interning				
No	1.042	0.889	1.223	0.611
Yes	1	-	-	-
Fear of contagion				
No fear	1.172	0.83	1.654	0.367
Little fear	0.782	0.602	1.017	0.066
Moderate fear	0.798	0.672	0.948	0.010^‡^
Severe fear	1	-	-	-
Coping strategies				
Physical activity				
Yes	1.009	0.836	1.218	0.924
No	1	-	-	-
Medication				
Yes	1.281	1.055	1.554	0.012^‡^
No	1	-	-	-
Psychotherapy				
Yes	1.238	1.053	1.456	0.010^‡^
No	1	-	-	-
Substance use				
Alcohol				
Yes	0.982	0.836	1.153	0.820
No	1	-	-	-
Medication				
Yes	0.741	0.602	0.91	0.004^†^
No	1	-	-	-
Place of residence				
Rural	1.187	1.019	1.383	0.028^‡^
Urban	1	-	-	-
Anxiety				
GAD-7 ≥10	2.039	1.658	2.507	< 0.001^§^
GAD-7 <10	1	-	-	-
Quality of life				
QLESQ	0.952	0.938	0.966	< 0.001^§^

95%CI = 95% confidence interval; GAD-7 = Generalized Anxiety Disorder 7-item; QLESQ = Quality of Life Enjoyment and Satisfaction Questionnaire.

Anxiety was defined as a Generalized Anxiety Disorder 7-item (GAD-7) score ≥ 10.

* Prevalence ratio.

^†^ p < 0.01; ^‡^ p < 0.05; ^§^ p < 0.001.

### Quality of life assessment

We used bivariate analysis to evaluate factors associated with impact on quality of life. Being female, having anxiety or depression, psychotherapy as a coping strategy, abusing alcohol, using medication (with or without a prescription), or fearing contagion were associated with worse quality of life scores. On the other hand, higher quality of life was associated with meditation or mindfulness practice and physical activity (Table S3, available as online-only supplementary material).

Multivariate analysis showed adopting meditation or mindfulness practices or physical activity as coping strategies were associated with higher quality of life among students on health care courses (twice and four times, respectively). The model also demonstrated that anxiety and depression were associated with lower quality of life (twice and six times, respectively). Moreover, fear of contagion (mild, moderate, or severe), suspected COVID-19, and use of medication (with or without a prescription) were associated with lower quality of life ( Table 4 ). In order to clarify the term used for medication, we defined taking medication with a medical prescription as a coping strategy and medication without a prescription as drug abuse.

**Table 4:** Multivariate Poisson regression model for quality of life in health care students (n = 462)

		95%CI		
			
Parameter	B*	Lower	Upper	p
Constant	50.753	46.709	54.797	< 0.001^†^
Undergraduate course				
Other courses	-0.359	-1.906	1.187	0.648
Pharmacy	1.374	-2.314	0.973	0.423
Nursing	-0.670	-0.264	3.011	0.100
Gender				
Female	-1.410	-2.867	0.046	0.058
Internship	0.780	-0.534	2.094	0.244
Fear of contagion				
Little fear	-5.998	-8.42	-3.576	< 0.001^†^
Moderate fear	-7.416	-9.531	-5.301	< 0.001^†^
Severe fear	-8.407	-10.753	-6.06	< 0.001^†^
Coping strategies				
Meditation/mindfulness	1.904	0.352	3.456	0.016^‡^
Physical activity	4.032	2.783	5.28	< 0.001^†^
Medication	-1.792	-3.372	-0.212	0.026^‡^
Psychotherapy	0.106	-1.252	1.464	0.878
Substance use				
Alcohol	-0.465	-1.759	0.828	0.480
Medication	-2.746	-4.606	-0.887	0.004^§^
Suspected COVID-19	-2.597	-1.172	-4.022	< 0.001^†^
Anxiety				
GAD-7 > 10	-2.301	-3.752	-0.85	0.002^§^
Depression				
PHQ-9 > 10	-5.993	-7.427	-4.559	< 0.001^†^

95%CI = 95% confidence interval; COVID-19 = coronavirus disease 2019; GAD-7 = Generalized Anxiety Disorder 7-item; PHQ-9 = Patient Health Questionnaire-9.

Quality of life was estimated using the Quality of Life Enjoyment and Satisfaction Questionnaire (QLESQ).

* Total effect of each variable on QLESQ.

^†^ p < 0.001; ^‡^ p <0.05; ^§^ p < 0.01.

### Comparison among undergraduate health courses

**Figure 1:**
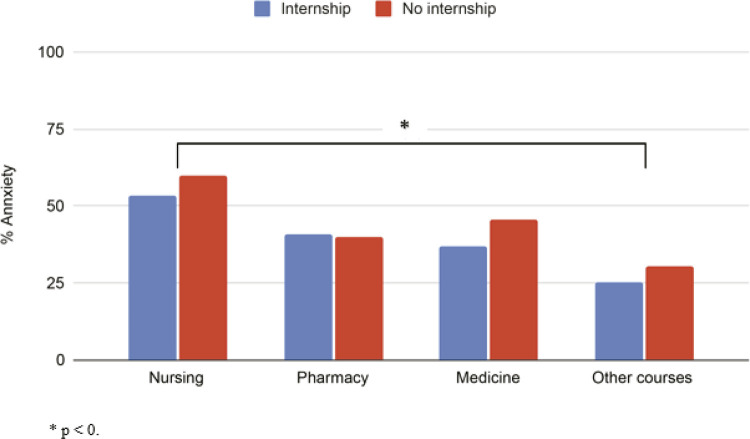
Percentage of students with severe anxiety comparing courses and internship status (n = 462). * p < 0.

When we compared GAD-7 scores by course, we observed that students from nursing courses had higher anxiety levels than the “other courses” group (53.8%, p = 0.005) ( Figure 1 ). Although more than 50% of the students were depressed, there were no statistically significant differences in PHQ-9 scores between the different health care programs. Other comparisons between student groups are described in Table S4 (online-only supplementary material).

## Discussion

In this study, we assessed mental health among Brazilian students on health care courses, searching for possible factors associated with anxiety and depressive symptoms, as a measure of psychiatric outcomes, and quality of life, as a parameter of overall functioning in this population. We identified factors associated with worsening of these psychiatric outcomes as well as factors that would potentially reduce some risks and improve the students’ quality of life.

The COVID-19 pandemic raised several concerns, such as doubts, fear, and social and personal isolation. For students on health care courses, there are concerns about risks, adaptations of their internship work, and different ways of dealing with the workplace and the world.^13 , 15 , 16 , 34^ In this pandemic situation, many universities closed and switched to online teaching and telehealth, causing students on health care courses distress.^6 , 14 , 35 , 36^ Despite the need for social isolation and the compromised healthcare learning, these students’ involvement as frontline health workers gave them some training and purpose. Given these circumstances, the impact of COVID-19 on mental health and habits is an increasingly important topic to consider.

We were able to identify higher rates of depression and anxiety in students on health care courses as compared with some previous studies using the same scales to screen for these disorders in other countries (China, Korea, Japan, United States, Ethiopia) during the pandemic.^9 , 15 , 35 , 37^ Students on health care courses are recognized as a vulnerable population, suffering from higher levels of anxiety and depression,^38^ but there seems to be a marked difference in the prevalence of these disorders in Brazilian students, compared to students in other countries.^15 , 39 - 41^ While the pooled prevalence estimates of anxiety and depression in Brazilian students from different undergraduate programs were 37.75 and 28.51%, respectively,^38^ in North American students, the prevalence of depression was 14.3-16% for medical students and 2.8% for students from different programs. Likewise, the prevalence of anxiety for North American students was 19% for medical students and 2.3% for students from different programs.^38 , 39 , 42^ Moreover, some of these studies investigated university students in general, not specifically those studying healthcare subjects.^35 , 38 , 43^ Non-healthcare students might be less prone to psychological problems compared with medical students.^44^

We identified associations between alcohol and medication abuse (no prescription) and psychiatric symptoms or low quality of life. In multivariate analyses, medication abuse was associated with greater anxiety and poorer quality of life. Recent studies in Brazilian, North American, and Ethiopian students suggested an increase in psychotropic drug consumption during the pandemic period, particularly among many undergraduate students and medical students.^34 , 35 , 38 , 45^ Our findings highlight the importance of these habits and their consequences for mental health. Moreover, fear of contagion was an important risk factor for students to have worse quality of life in their work. The increase in consumption of alcohol and fear of contagion prompts us to rethink some aspects of mental distress, coping, and behaviors during the pandemic.^35 , 46 , 47^

In contrast to these adverse factors, some strategies have already been described as protective factors against stressful environments and anxiety.^3 , 18 , 48 - 54^ In our multivariate analysis, psychotherapy was a potential coping strategy for anxiety. Many studies indicate that cognitive behavioral therapy is an effective technique for anxiety disorders,^55 - 59^ not only in face-to-face settings but also in online formats.^60 , 61^ On the other hand, psychotherapy was associated with presence of depression in the multivariate analysis and with poor quality of life in bivariate analyses, although this was not confirmed in multivariate analyses. Possibly, this association could be considered a consequence of the presence of symptoms and an initial search for treatment in depressive students.^18 , 62^ The cross-sectional design of our study cannot support conclusions about causality. Other effective coping strategies identified in our study were meditation or mindfulness practice and physical activity, which greatly enhanced resilience and improved the students’ quality of life. These procedural strategies are important protective factors for mental health.^52 , 63 - 66^

Whereas some studies in Brazil, the United States, and India have discussed the presence of high rates of anxiety and depression in medical students,^10 , 12 , 15 , 34 , 67 , 68^ there are very few data on students from other health care programs.^34 , 67^ We therefore compared differences between participants on medical, nursing, and pharmacy courses, and on other courses. We found a higher prevalence of anxiety in nursing students, and a higher rate of exposure to internship in this group, corroborating published studies that address mental health problems and low quality of life in Brazilian and North American nursing students.^12 , 16 , 34 , 69 , 70^ Interestingly, multivariate analysis demonstrated that the highest risk for anxiety was related to the nursing course. Studies indicate that anxiety can have a negative effect on these students’ quality of life of, both in training and in clinical practice,^71^ and can even cause them to drop out of the nursing program.^72^ Consistently, a Spanish study reported that final nursing graduate students felt highly committed to their internships during the COVID-19 pandemic, reporting more anxiety than usual.^10^

Another important question that should be considered is the impact of stigma on the mental health of the population affected, especially healthcare workers and students.^73 , 74^ People with current or past COVID-19 and their relatives, social minorities, and healthcare workers have experienced COVID-19 related stigma. These populations could be suffering a range of stigma experiences and practices that can reduce their social support and damage their mental health. Policies and actions to reduce the impacts of COVID-19 related stigma, such as education of the general population and media, celebration of those at the forefront of the pandemic, and fighting myths and misinformation, are very important to protect healthcare workers and students.^70 , 73 , 74^

This study has some limitations that should be acknowledged. First, we adopted an online convenience sampling strategy, which was not based on a random selection. Although the results are from students on health care courses, it is difficult to generalize the results to those students who do not have access to social networks. However, considering the pandemic context, we believe this is the most viable strategy. Second, sample variability was limited to students from a few universities across the country and the sample does not represent the totality of Brazilian students. Third, this study was performed on a limited group of courses, which makes it difficult to generalize our results to other courses. Fourth, the majority of participants were female, and the possibility of sampling bias should be considered, nevertheless gender differences detected were considered in multivariate analyses. Fifth, participants who used prescribed medications were not asked whether they had received a diagnosis prior to the pandemic or what medications they use, and these questions could affect the results. Finally, since this is a cross-sectional study, causal inferences cannot be effectively demonstrated.

On the other hand, our study provides important information about the factors and coping strategies associated with emotional distress, symptoms, and quality of life that could help design appropriate mental health coping interventions. Thereby, this study can further corroborate in future research on some psychological aspects of the health care student population.

## Conclusion

The rapid spread of COVID-19 is associated with distress among students on health care courses. The pandemic has had a significant adverse impact on mental health and influenced lifestyle. Our study showed that the COVID-19 pandemic may affect the mental health and well-being of students on health care courses. We also present important information about the factors and coping strategies associated with psychological impacts of the COVID-19 pandemic that should be helpful for designing appropriate mental health interventions. It was observed that nursing students had a higher rate of psychological problems than students on pharmacy, physiotherapy, medicine, and other health courses. Therefore, there is a need to help students to deal with this stressful event, supporting development of self-care practices among students and taking special care with regard to psychological outcomes in order to enable students on health care courses to be more resilient during times of uncertainty.
