# Supplementary material for: Psychiatric outcomes and overall functioning in healthcare students during the first wave of the COVID-19 pandemic: a cross-sectional study
Source: Trends Psychiatry Psychother. 2023 Jun 27;45:e20210416. doi: 10.47626/2237-6089-2021-0416 (PMC10416255; doi:10.47626/2237-6089-2021-0416)
Supplement: Supplementary file 1 [file 2238-0019-trends-45-e20210416-suppl1.pdf]

Supplementary Material S1

Convite para participar de pesquisa

Você está sendo convidado a participar de uma pesquisa que tem por objetivo avaliar o impacto da pandemia da COVID-19 sobre as tarefas cotidianas e acadêmicas, bem como avaliar os sintomas relacionados ao estresse, à ansiedade, à depressão e à qualidade de vida em estudantes de diferentes cursos da área da saúde que estão atuando no enfrentamento da pandemia do coronavírus, através da aplicação de questionários e instrumentos psicométricos autoaplicáveis na forma online. Se você tem interesse em participar da pesquisa clique aqui e você será direcionado (a) para o Termo de Consentimento Livre e Esclarecido, documento que contém mais informações sobre a pesquisa. A participação na pesquisa será por meio de resposta a um questionário sobre como está lidando com questões acadêmicas, sociais e emocionais durante seu estágio acadêmico no período de pandemia. Se após a leitura do Termo de Consentimento você decidir participar da pesquisa, responda à pergunta: Você concorda em participar da pesquisa? Ao responder Sim você será direcionado para o questionário. O tempo médio de resposta é de 7 minutos.
Agradecemos o seu tempo e atenção.
Equipe de pesquisa.

Convite para as mídias sociais

Precisamos de sua ajuda.
Ultimamente temos lido e ouvido muito sobre questões dos pacientes e dos profissionais da saúde, mas queremos saber de VOCÊ, estudante da saúde que está atuando no enfrentamento da pandemia da COVID-19.
Nós do Programa de Transtornos de Ansiedade do Hospital de Clínicas de Porto Alegre (PROTAN), queremos avaliar o impacto nas tarefas cotidianas e acadêmicas, estratégias de *coping*, sintomas relacionados ao estresse, à ansiedade, à depressão e a qualidade de vida em estudantes da área da saúde que estão atuando no enfrentamento da pandemia do coronavírus.

Table S1 - Differences between anxious and non-anxious students (n = 462)

	**No anxiety (n = 280)**	**Anxiety (n = 182)**	
	**(GAD-7 < 10)**	**(GAD-7 ≥ 10)**	**p-value**
Gender, n (%)			
Female	216 (77.1)	153 (84.1)	0.070
Male	64 (22.9)	29 (15.9)	
Age, median (minimum-maximum)	23 (16-61)	23 (19-43)	0.177
Quality of life (QLESQ), mean (SD)	47.08 (7.78)	39.05 (7.42)	< 0.001*
Depression, n (%)			
PHQ-9 < 10	198 (70.7)	26 (14.3)	<0.001*
PHQ-9 ≥ 10	82 (29.3)	156 (85.7)	
Place of residence, n (%)			
Urban	199 (71.1)	110 (60.4)	0.018†
Rural	81 (28.9)	72 (39.6)	
Internship, n (%)			
No	78 (27.9)	51 (28.0)	0.969
Yes	202 (72.1)	131 (72.0)	
Living alone, n (%)			
No	239 (85.4)	148 (81.3)	0.250
Yes	41 (14.6)	34 (18.7)	
Coping strategies, n (%)			
Diaphragmatic breathing	99 (35.4)	48 (26.4)	0.043†
Progressive muscle relaxation	24 (8.6)	14 (7.7)	0.737
Meditation/mindfulness	46 (16.4)	29 (15.9)	0.888
Yoga	25 (8.9)	16 (8.8)	0.960
Physical activity	105 (37.5)	45 (24.7)	0.004‡
Medication	48 (17.1)	59 (32.4)	< 0.001*
Psychotherapy	79 (28.2)	47 (25.8)	0.573
Substance use, n (%)			
Alcohol	67 (23.9)	66 (36.3)	0.004‡
Medication	19 (6.8)	48 (26.4)	< 0.001*
Cannabis	14 (5.0)	11 (6.0)	0.628
Cocaine	2 (0.7)	2 (1.1)	0.648
Suspected COVID-19 (%)			
No	228 (81.4)	138 (75.8)	0.147
Yes	52 (18.6)	44 (24.2)	
Fear of contagion (%)			
Severe fear	46 (16.4)§	53 (29.1)‖	0.001‡
Moderate fear	150 (53.6)§	97 (53.3)§	
Little fear	51 (18.2)§	22 (12.1)§	
No fear	33 (11.8)§	10 (5.5)‖	

COVID-19 = coronavirus disease 2019; GAD-7 = Generalized Anxiety Disorder 7-item;
PHQ-9 = Patient Health Questionnaire-9; QLESQ = Quality of Life Enjoyment and Satisfaction Questionnaire; SD = standard deviation.
Chi-square test was applied for testing categorical variable differences between anxiety status. Independent samples Mann-Whitney *U* sum test was used to test differences of age between groups. Independent samples *T* test was used to examine differences of quality of life (QLESQ) between groups.
* p < 0.001; † p < 0.05; ‡ p < 0.01; §‖ Each symbol denotes a subset of categories whose column proportions do not differ significantly from each other at the 0.05 level.

Table S2 - Differences between depressive and non-depressive students (n = 462)

	**No depression (n=224)** (PHQ-9 < 10)	**Depression (n=238)** (PHQ-9 ≥ 10)	**p-value**
Gender, n (%)			
Female	168 (75.0)	201 (84.5)	0.011*
Male	56 (25.0)	37 (15.5)	
Age, mean (SD)	24 (16-61)	23 (19-61)	0.009*
Quality of life (QLESQ), mean (SD)	48.91 (7.5)	39.22 (6.69)	< 0.001†
Anxiety, n (%)			
GAD-7 < 10	198 (70.7)	26 (14.3)	< 0.001†
GAD-7 ≥ 10	82 (29.3)	156 (81.7)	
Place of residence, n (%)			
Urban	166 (74.1)	143 (60.1)	0.001‡
Rural	58 (25.9)	95 (39.9)	
Internship, n (%)			
No	61 (27.2)	68 (28.6)	0.748
Yes	163 (72.8)	170 (71.4)	
Living alone, n (%)			
No	192 (85.7)	195 (81.9)	0.271
Yes	32 (14.3)	43 (18.1)	
Coping strategies, n (%)			
Diaphragmatic breathing	79 (35.3)	68 (28.6)	0.122
Progressive muscle relaxation	17 (7.6)	21 (8.8)	0.629
Meditation/mindfulness	39 (17.4)	36 (15.1)	0.506
Yoga	16 (7.1)	25 (10.5)	0.204
Physical activity	87 (38.8)	63 (26.5)	0.005‡
Medication	24 (10.7)	83 (34.9)	< 0.001†
Psychotherapy	47 (21.0)	79 (33.2)	0.003‡
Substance use, n (%)			
Alcohol	54 (24.1)	79 (33.2)	0.031*
Medication	14 (6.3)	53 (22.3)	< 0.001†
Cannabis	13 (5.8)	12 (5.0)	0.718
Cocaine	0 (0.0)	4 (1.7)	0.124
Suspected COVID-19 (%)			
No	185 (82.6)	181 (76.1)	0.083
Yes	39 (17.4)	57 (23.9)	
Fear of contagion (%)			
Severe fear	27 (12.1)§	72 (30.3)§	< 0.001†
Moderate fear	128 (57.1)ǁ	119 (50.0)ǁ	
Little fear	43 (19.2)ǁ	30 (12.6)ǁ	
No fear	26 (11.6)ǁ	17 (7.1)ǁ	

COVID-19 = coronavirus disease 2019; GAD-7 = Generalized Anxiety Disorder 7-item; PHQ-9 = Patient Health Questionnaire-9; QLESQ = Quality of Life Enjoyment and Satisfaction Questionnaire; SD = standard deviation.
Chi-square test was applied for testing categorical variable differences between anxiety status. Independent samples Mann-Whitney *U* sum test was used to test differences of age between groups. Independent samples *t* test was used to examine differences of quality of life (QLESQ) between groups.
* p < 0.05; † p < 0.001; ‡ p < 0.01; §ǁ Each symbol denotes a subset of categories whose column proportions do not differ significantly from each other at the 0.05 level.

Table S3 - Associations between variables and quality of life in health students (n = 462)

	**Quality of life, mean (SD)**	**p-value**
Gender		
Female	43.33 (8.10)	0.01*
Male	46.25 (9.99)	
Anxiety		
GAD-7 < 10	47.08 (7.78)	< 0.001†
GAD-7 ≥ 10	39.05 (7.42)	
Depression		
PHQ-9 < 10	48.91 (7.50)	< 0.001†
PHQ-9 ≥ 10	39.22 (6.68)	
Place of residence		
Urban	44.36 (8.72)	0.117
Rural	43.03 (8.26)	
Internship		
No	44.52 (8.57)	0.347
Yes	43.68 (8.59)	
Living alone		
No	43.85 (8.59)	0.699
Yes	44.27 (8.57)	
Coping strategies		
Diaphragmatic breathing		
No	43.62 (8.92)	0.255
Yes	44.55 (7.81)	
Progressive muscle relaxation		
No	43.91 (8.72)	0.934
Yes	44.03 (6.98)	
Meditation/mindfulness		
No	43.51 (8.63)	0.021*
Yes	46.01 (8.07)	
Yoga		
No	43.74 (8.60)	0.151
Yes	45.76 (8.29)	
Physical activity		
No	42.24 (8.26)	< 0.001†
Yes	47.39 (8.22)	
Medication		
No	45.13 (8.29)	< 0.001†
Yes	39.89 (8.33)	
Psychotherapy		
No	44.45 (8.78)	0.029*
Yes	42.49 (7.88)	
Substance use		
Alcohol		
No	44.67 (8.45)	0.003‡
Yes	42.05 (8.66)	
Medication		
No	44.97 (7.91)	< 0.001†
Yes	37.67 (9.71)	
Cannabis		
No	43.95 (8.51)	0.704
Yes	43.28 (9.99)	

Cocaine		
No	43.91 (8.53)	0.984
Yes	44.00 (15.03)	
Suspected COVID-19		
No	44.64 (7.81)	0.003‡
Yes	41.14 (10.64)	
Fear of contagion		
Severe fear	40.11§ (7.77)	< 0.001†
Moderate fear	43.51‖ (7.68)	
Little fear	45.95‖ (8.47)	
No fear	51.53¶ (9.88)	

COVID-19 = coronavirus disease 2019; GAD-7 = Generalized Anxiety Disorder 7-item;
PHQ-9 = Patient Health Questionnaire-9; QLESQ = Quality of Life Enjoyment and Satisfaction Questionnaire; SD = standard deviation.
Independent samples *t* test was used to examine differences of quality of life (QLESQ) between groups.
* $p < 0.05$; † $p < 0.001$; ‡ $p < 0.01$; §‖¶ Each symbol denotes a subset of categories whose column proportions do not differ significantly from each other at the 0.05 level.

Table S4 - Differences among undergraduate courses (n = 462)

	Nursing (n = 80)	Pharmacy (n = 84)	Medicine (n = 197)	Other courses (n = 101)	p-value
Gender, n (%)					
Female	68 (85.0)	67 (79.8)	153 (77.7)	81 (80.2)	0.591
Male	12 (15.0)	17 (20.2)	44 (22.3)	20 (19.8)	
Age, mean (SD)	24.35 (5.03)	26.46 (7.42)	24.33 (4.69)	26.12 (7.40)	0.091
Anxiety, n (%)					
GAD-7 < 10	37 (46.3)*	50 (59.5)*†	120 (60.9)*†	73 (72.3)†	0.005‡
GAD-7 ≥ 10	43 (53.8)*	34 (40.5)*†	77 (39.1)*†	28 (27.7)†	
Depression, n (%)					
PHQ-9 < 10	35 (43.8)	48 (57.1)	93(47.2)	48 (47.5)	0.333
PHQ-9 ≥ 10	45 (56.3)	36 (42.9)	104 (52.8)	53 (52.5)	
Quality of life (QLESQ), mean (SD)	42.51 (8.62)	45.35 (8.26)	44.09 (8.84)	43.50 (8.23)	0.189
Place of residence, n (%)					
Urban	58 (72.5)	55 (65.5)	122 (61.9)	74 (73.3)	0.154
Rural	22 (27.5)	29 (34.5)	75 (38.1)	27 (26.7)	
Internship, n (%)					
No	5 (6.3)*	30 (35.7)†§	48 (24.4)§	46 (45.5)†	< 0.001‖
Yes	75 (93.8)*	54 (64.3)†§	149 (75.6)§	55 (54.5)†	
Living alone, n (%)					
No	67 (83.8)	72 (85.7)	157 (79.7)	91 (90.1)	0.132
Yes	13 (16.3)	12 (14.3)	40 (20.3)	10 (9.9)	
Coping strategies, n (%)					
Diaphragmatic breathing	18 (22.5)*	24 (28.6)*†	63 (32.0)*†	42 (41.6)†	0.045¶
Progressive muscle relaxation	8 (10.0)*†	2 (2.4)†	14 (7.1)*†	14 (13.9)*	0.033¶
Meditation/mindfulness	11 (13.8)	13 (15.5)	38 (19.3)	13 (12.9)	0.459
Yoga	7 (8.8)	3 (3.6)	23 (11.7)	8 (7.9)	0.176
Physical activity	26 (32.5)*†	16 (19.0)†	68 (34.5)*†	40 (39.6)*	0.022¶
Medication	18 (22.5)	22 (26.2)	49 (24.9)	18 (17.8)	0.495
Psychotherapy	25 (31.3)*	9 (10.7)†	56 (28.4)*	36 (35.6)*	0.001‡

Substance use, n (%)					
Alcohol	25 (31.3)*†	14 (16.7)†	68 (34.5)*	26 (25.7)*†	0.020¶
Medication	7 (8.8)	14 (16.7)	33 (16.8)	13 (12.9)	0.324
Cannabis	4 (5.0)	4 (4.8)	12 (6.1)	5 (5.0)	0.957
Cocaine	0 (0.0)	3 (3.6)	1 (0.5)	0 (0.0)	0.072
Suspected COVID-19 (%)					
No	61 (76.3)	74 (88.1)	148 (75.1)	83 (82.2)	0.072
Yes	19 (23.8)	10 (11.9)	49 (24.9)	18 (17.8)	
Fear of contagion (%)					
Severe fear	17 (21.3)*	15 (17.9)*	38 (19.3)*	29 (28.7)*	0.003‡
Moderate fear	46 (57.5)*	45 (53.6)*	97 (49.2)*	59 (58.4)*	
Little fear	7 (8.8)*	18 (21.4)*	35 (17.8)*	13 (12.9)*	
No fear	10 (12.5)*	6 (7.1)*	27 (13.7)*	0 (0.0)†	

COVID-19 = coronavirus disease 2019; GAD-7 = Generalized Anxiety Disorder 7-item;
PHQ-9 = Patient Health Questionnaire-9; QLESQ = Quality of Life Enjoyment and Satisfaction Questionnaire; SD = standard deviation.
Chi-square test was applied for testing categorical variable differences between undergraduate course groups. Kruskal-Wallis rank sum test was used to test differences of age between groups. One-way analysis of variance (ANOVA) and Tukey post-hoc test was used to examine differences of quality of life (QLESQ) between groups.
*†§ Each symbol denotes a subset of categories whose column proportions do not differ significantly from each other at the 0.05 level.
‡ $p < 0.01$; ǁ $p < 0.001$; ¶ $p < 0.05$.
